# BRIE: transcriptome-wide splicing quantification in single cells

**DOI:** 10.1186/s13059-017-1248-5

**Published:** 2017-06-27

**Authors:** Yuanhua Huang, Guido Sanguinetti

**Affiliations:** 1 School of Informatics, University of Edinburgh, Edinburgh, EH8 9AB UK; 2 Centre for Synthetic and Systems Biology (SynthSys), University of Edinburgh, Edinburgh, EH9 3BF UK

**Keywords:** Single-cell RNA-seq, Isoform estimate, Differential splicing

## Abstract

Single-cell RNA-seq (scRNA-seq) provides a comprehensive measurement of stochasticity in transcription, but the limitations of the technology have prevented its application to dissect variability in RNA processing events such as splicing. Here, we present BRIE (Bayesian regression for isoform estimation), a Bayesian hierarchical model that resolves these problems by learning an informative prior distribution from sequence features. We show that BRIE yields reproducible estimates of exon inclusion ratios in single cells and provides an effective tool for differential isoform quantification between scRNA-seq data sets. BRIE, therefore, expands the scope of scRNA-seq experiments to probe the stochasticity of RNA processing.

**Electronic supplementary material:**

The online version of this article (doi:10.1186/s13059-017-1248-5) contains supplementary material, which is available to authorized users.

## Background

Next-generation sequencing technologies have revolutionized our understanding of RNA biology, illustrating both the diversity of the transcriptome and the richness and complexity of the regulatory processes controlling transcription and RNA processing. Recently, efficient RNA amplification techniques have been coupled with next-generation sequencing to yield transcriptome sequencing protocols for measuring the abundance of transcripts within single cells, known as single-cell RNA-seq (scRNA-seq) [1]. scRNA-seq has provided unprecedented opportunities to investigate the stochasticity of transcription and its importance in cellular diversity. Groundbreaking applications of scRNA-seq include the ability to discover novel cell types [2], to study transcriptome stochasticity in response to external signals [3], and to enhance cancer research by dissecting tumor heterogeneity [4], to mention but a few. However, such advances have been limited to exploring the variability between single cells at the gene level, and we know very little about the global variability of RNA splicing between individual cells. Bulk RNA-seq splicing quantification algorithms cannot be easily adapted to the single-cell case due to the minute amounts of starting material, low cDNA conversion efficiency, and uneven transcript coverage resulting in intrinsically low coverage and potentially high technical noise [5]. This considerably limits the usefulness of scRNA-seq to investigating questions about RNA processing and splicing at the single-cell level.

Splicing analysis has been revolutionized by the advent of (bulk) RNA-seq techniques. Early studies [6] quantified splicing by considering junction reads that are uniquely assigned to an inclusion/exclusion isoform, necessitating very high coverage depth to achieve confident predictions. The situation can be considerably improved by using probabilistic methods based on mixture modeling, an idea that is at the core of standard tools such as Cufflinks [7] and MISO [8]. Nevertheless, low coverage represents a challenge even for probabilistic methods. Recent work has shown that improved predictions at lower coverage can be achieved by incorporating informative prior distributions within probabilistic splicing quantification algorithms, leveraging either aspects of the experimental design, such as a time series [9], or auxiliary data sets, such as measurements of PolII localization [10]. Such auxiliary data are not normally available for scRNA-seq data. Nevertheless, recent studies have also demonstrated that splicing (in bulk cells) can be accurately predicted from sequence-derived features [11]. This suggests that overall patterns of read distribution may be associated with specific sequence words, so that one may be able to construct informative prior distributions learned directly from data. Here we introduce the Bayesian regression for isoform estimation method (BRIE), a statistical model that achieves extremely high sensitivity at low coverage by using informative priors learned directly from data via a (latent) regression model. The regression model couples the task of splicing quantification across different genes, allowing a statistical transfer of information from well-covered genes to less well covered genes, achieving considerable robustness to noise in low coverage.

## Results and discussion

### High-level model description

**Fig. 1:**
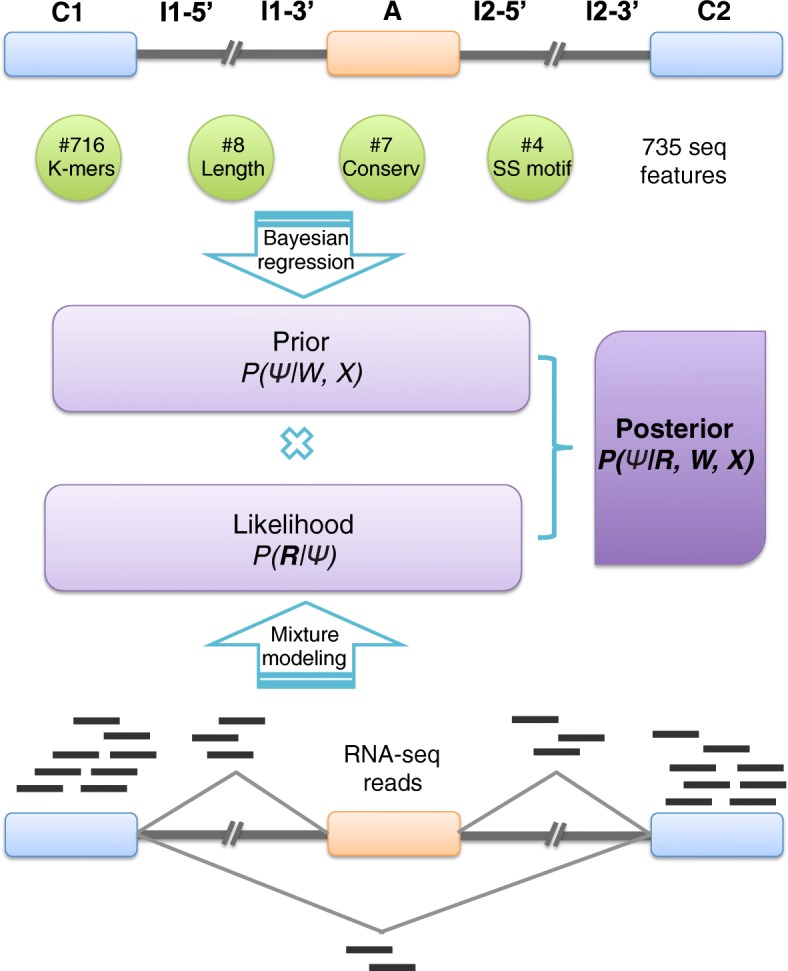
A cartoon of the BRIE method for isoform estimation. BRIE combines a likelihood computed from RNA-seq data (*bottom part*) and an informative prior distribution learned from 735 sequence-derived features (*top*)

Figure 1 presents a schematic illustration of BRIE (see “Methods” section for precise definitions and details of the estimation procedure). The bottom part of the figure represents the standard mixture model approach to isoform estimation introduced in MISO [8] and Cufflinks [7], where reads are associated with a latent, multinomially distributed isoform identity variable (see “Methods” section for a self-contained review of mixtures of isoforms models). This module takes as input the scRNA-seq data (aligned reads) and forms the likelihood of our Bayesian model. The multinomial identity variables are assigned an informative prior in the form of a regression model (top half of Fig. 1), where the prior probability of inclusion ratios is regressed against sequence-derived features. Crucially, the regression parameters are shared across all genes and can be learned across multiple single cells, thus regularizing the task and enabling robust predictions in the face of very low coverage. In the “Methods” section and supplementary material, we give details of the features used. While the class of regression models we employ is different from the neural networks of [11], they still provide a highly accurate supervised learning predictor of splicing on bulk RNA-seq data sets. Additional file 1: Figure S1 shows that the Bayesian regression approach of BRIE can achieve a Pearson *R* in excess of 0.8 on test sets, validating our choice of model within BRIE.

This architecture effectively enables BRIE to trade off two tasks simultaneously: in the absence of data (drop-out genes), the informative prior provides a way of imputing missing data, while for highly covered genes the likelihood term dominates, returning a mixture-model quantification. For intermediate levels of coverage, BRIE uses Bayes’s theorem to trade off imputation and quantification.

### Benchmarking BRIE on simulated data

To assess the improvement in isoform quantification afforded by BRIE’s informative prior, we simulated RNA-seq reads for 11,478 human exon-skipping events, and a correlated feature to learn the prior (see details in “Methods” section and Additional file 1: Figure S2). As we are interested in quantifying the effects of an informative prior, we compare BRIE with similar methods developed for bulk RNA-seq: MISO v0.5.3 [8], one of the first and still very widely used probabilistic methods, and DICE-seq v0.2.6 [9], a modification of MISO using informative priors (for multiple time points). For completeness, we also compare with Kallisto [12], which was recently proposed as one of the most computationally efficient and robust quantification tools. To simulate the effect of the regression prior, we introduced an auxiliary variable with correlation 0.8 with the desired inclusion ratios (the correlation value was chosen to match the empirical performance of BRIE’s regression prior on bulk RNA-seq data in Additional file 1: Figure S1). We also consider the case when BRIE’s auxiliary variable is uncorrelated with the inclusion ratio (denoted as BRIE.Null) as a control. Thanks to the informative prior, BRIE can also provide an imputation for drop-out transcripts (see below), which other methods cannot; to keep the simulation fair, we did not include results on drop-out genes.

**Fig. 2:**
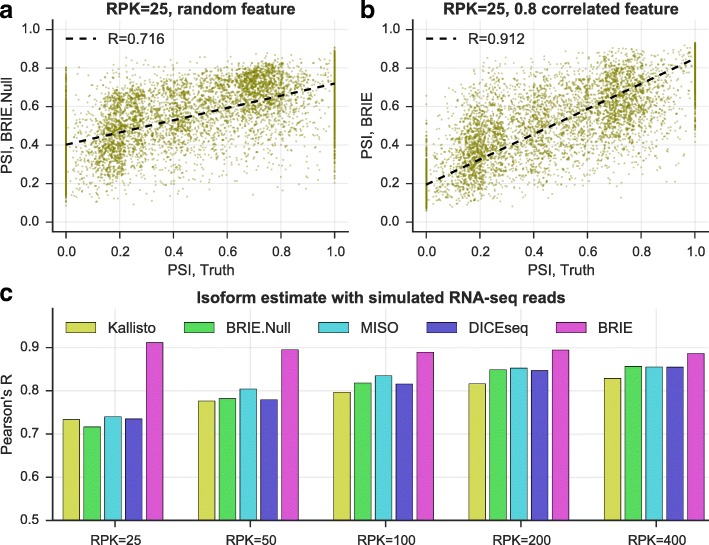
BRIE improves isoform estimates by using an informative prior on simulated data. **a, b** At very low coverage RPK=25, a scatter plot between the estimates of the exon inclusion ratios by BRIE and the simulation truth. **a** BRIE.Null uses five random uniformly distributed features to learn the prior. **b** BRIE uses one correlated feature with Pearson’s *R*=0.8 to the truth to learn an informative prior. **c** Pearson’s *R* between truth and estimate by BRIE, BRIE.Null, and three other methods for different coverages. *RPK* reads per kilobase

In the simulation, we set different coverage levels with RPK (reads per kilobase) ranging from 25 to 400. Figure 2 clearly shows that an informative prior can bring very substantial performance improvements at low coverage. At the lowest RPK level, BRIE achieves a gain of almost 20 % in correlation between estimates and ground truth. Furthermore, this accuracy level is essentially maintained by BRIE at all coverage values. Interestingly, BRIE.Null can still achieve comparable accuracy to the other methods at all coverage values. Therefore, even when an informative prior could not be effectively learned, BRIE’s results would not be worse than using a state-of-the-art bulk RNA-seq method.

### Imputation of drop-out in simulation

The informative prior learned by BRIE can also be used to impute isoform usage when there is a drop-out, i.e., when no reads are sequenced for an expressed isoform. In scRNA-seq experiments, drop-outs occur widely [5], though they are sometimes hard to detect exactly, except for spike-in RNAs. Here, we could coarsely define the upper bound by counting exon-skipping events expressed in bulk cells but not in a given single cell. In Additional file 1: Figure S3, we see that after removing drop-out events, the correlation of expression levels between a single cell and bulk cells are dramatically higher for these splicing events.

As BRIE can transfer information from a highly expressed gene to lowly expressed genes across multiple cells, we investigated the performance of BRIE in imputing the isoform usage if a drop-out happens. Therefore, the expression profile from a bulk RNA-seq library and the drop-out probability profile estimated from 96 HCT116 human cell scRNA-seq libraries [13] (see Additional file 1: Figure S4) were used for the simulation (see simulation details in “Methods” section). Additional file 1: Figure S5 shows that BRIE can produce a good imputation of the isoform usage simply by taking the mean of the informative prior learned from sequence features of the expressed genes (Pearson’s *R*=0.6–0.7).

### BRIE yields robust splicing estimates on real data

**Fig. 3:**
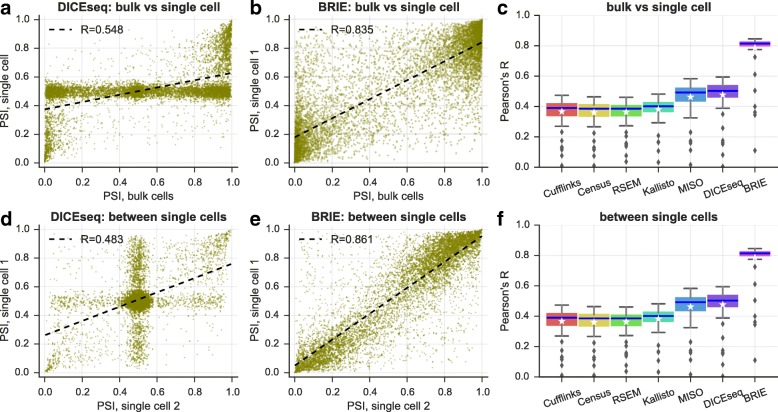
BRIE improves splicing estimates by using sequence features. **a–c** Pearson’s correlation between bulk and single cells on exon inclusion ratio *ψ* in HCT116 cells. Scatter plot of *ψ* estimates by DICEseq (**a**) or estimated by BRIE (**b**). Box plot for all methods (**c**) in 96 cells. **d–f** Pearson’s correlation between single-cell pairs. Scatter plot of *ψ* estimates by DICEseq (**d**) or estimated by BRIE (**e**). Box plot for all methods (**f**) in 4608 cell pairs

To assess BRIE’s performance on real scRNA-seq data, we used 96 scRNA-seq libraries from individual HCT116 human cells from the benchmark scRNA-seq study of Wu et al. [13] (see “Methods” section for details). Importantly, a bulk RNA-seq data set in the same conditions was also obtained from one million cells. To explore performance on real data better, we expanded the set of competing methods to include Cufflinks v2.2.1 [7], RSEM v1.3.0, and the recently proposed single-cell quantification method Census (in Monocle v2.2.0) based on Cufflinks Fragments Per Kilobase per Million (FPKM) [14]. Figure 3 shows the results: BRIE clearly outperforms all other methods by a large margin, both in terms of correlation between estimates from different single cells (Fig. 3f), and in terms of correlations between estimates from individual single cells and bulk (Fig. 3c). Example scatter plots for both comparisons are given in Fig. 3e and b, clearly showing very consistent predictions. Notably, the performance of other methods was strongly degraded by the inability to handle the large drop-out rates (see Fig. 3a and d for DICE-seq, where many estimates of splicing are centered around the uninformative prior value of 0.5). The high correlation between bulk and scRNA-seq predictions is particularly remarkable, as the analysis of the two data sets is not done with a shared prior. Similar levels of correlation were found between splicing estimates obtained by BRIE in single cells and estimates from bulk RNA-seq obtained by other methods (Additional file 1: Figure S6).

These statistical advantages are reflected in a more effective and confident quantification: considering genes with quantified uncertainty smaller than 0.3 (a threshold adopted, e.g., in [15] to select for downstream analysis), Additional file 1: Figure S7 shows that BRIE retained 10.9 % out of 11,478 genes on average from each single cell, compared with 3.1 % and 5.6 % for MISO and DICE-seq, respectively.

### BRIE gives higher sensitivity in differential splicing analyses

BRIE can also be used for differential splicing detection across different data sets. To do so, we compute the evidence ratio (Bayes factor, BF) between a model where the two data sets are treated as replicates (null hypothesis) and an alternative model where the two data sets are treated as separate. We use the Savage–Dickey density-ratio approach and relax it to obtain more robust estimates (see “Methods” section). Notice that there are several ways in which differential comparisons could be performed: we could compare groups of cells or individual cells, and we could share the learning of the prior across conditions, or learn separately. All of these options are supported in the BRIE software.

To benchmark the effectiveness of this strategy, we again turned to a simulation study, investigating the ability of BRIE to detect differential splicing as we vary coverage and the extent of the differential effect (see “Methods” section for details of the simulation). This benchmarking is important, as the informative prior might be expected to impede differential quantification. In practice, we see that, for substantial effect sizes (*Δ* *ψ*=0.6), we can detect a substantial fraction of differentially spliced genes already at 50 RPK, further improving when the effect size is 0.8 (Additional file 1: Figure S8a). We also use the simulation to explore the effect of different library sizes on our differential comparisons. We do this by fixing one of the comparison cells to an RPK level. The results shown in Additional file 1: Figure S8b, c demonstrate that BRIE is robust to normalization issues. This is not surprising, since relative quantification algorithms normally combine normalization with estimation (see [14] for a discussion of this topic in the scRNA-seq context).

**Fig. 4:**
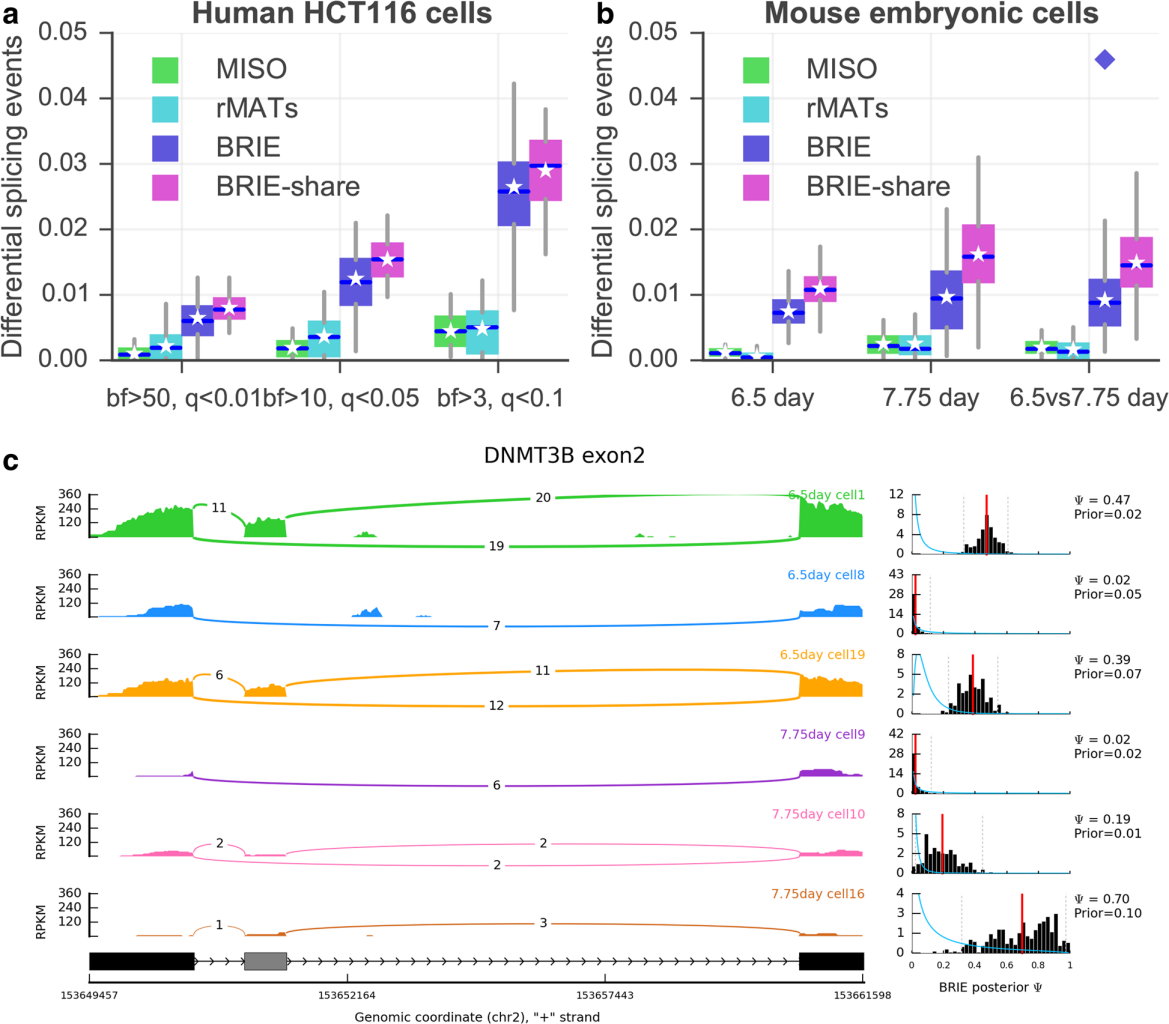
Detection of differential splicing between cells. **a** Percentage of differential splicing events between human HCT116 cells, detected by MISO, rMATS, BRIE, and its mode with shared weights (i.e., BRIE.share) with different thresholds. MISO and BRIE use the Bayes factor (BF) and rMATS uses false discovery rate (*q* value). **b** Percentage of differential splicing events between mouse early embryonic cells at day 6.5 or day 7.75. The threshold is BF>10 for MISO and BRIE, and *q*<0.05 for rMATS. The *diamond* indicates pooled reads of 20 cells in each group. **c** An example exon-skipping event in DNMT3B in three mouse cells at 6.5 days and three cells at 7.75 days. The *left panel* is a sashimi plot of the read density and the number of junction reads. The *right panel* shows the prior distribution as a *blue curve* and a histogram of the posterior distribution in *black*, both learned by BRIE. For the histogram, the *red line* is the mean and the *dashed lines* are the 95 % confidence interval. *BF* Bayes factor

We then investigated the effectiveness of BRIE to detect differential splicing in real cells. To estimate a background level of differential splicing between identical cells, we considered again the 20 single-cell HCT116 libraries from Wu et al. [13], and compared all possible pairs of cells. Figure 4a shows the fraction of genes called as differentially spliced at different BF thresholds in this control experiment. As we can see, this number is always very small, and around 1 % at the normally recommended threshold of BF=10. This level of background calling could be partly attributed to intrinsic stochasticity or to residual physiological variability that was not controlled for in the experiment, such as cell cycle phase. As an additional comparison, we considered two bulk RNA-seq methods for differential splicing, MISO and the recently proposed rMATS [16]. Both methods could call only a negligible number of events, far fewer than the expected number of false positives, confirming that bulk methods are not suitable for scRNA-seq splicing analysis.

We then considered a mouse early development scRNA-seq data set [17], and compared the single-cell transcriptomic profiles from cells from mouse embryos at 6.5 and 7.75 days. We compared both the profiles of individual cells at the same and different time points. The results are summarized in Fig. 4b. Comparing individual cells at 6.5 days yielded approximately 1 % of events called as significantly differential (BF ≥10) at 6.5 days. Comparing this result with our investigation of HCT116 cells suggests that murine cells at 6.5 days are still like a homogeneous population, from the splicing point of view. The percentage nearly doubled at 7.75 days, suggesting that differential splicing becomes more widespread at this later stage of differentiation. A similar fraction of exon-skipping events were differentially called between cells at 7.75 days and cells at 6.5 days. To define a group of differentiation-associated skipping events, we considered events that we called as differential in at least 10 % of 7.75 vs 6.5 comparisons. The resulting 159 events were highly enriched for organelle and intracellular part gene ontology terms (*p*<0.01) (see Additional file 2: Tables S1 and S2). Figure 4c shows the example of DNMT3B, a regulator of DNA methylation maintenance, which is known to undergo functionally relevant alternative splicing [18]. DNMT3B exhibited differential splicing between 7.75 days and 6.5 days in 153 out of 400 comparisons between individual single cells, clearly highlighting the strong differential inclusion effect. Four more example events, all of which show differential splicing in more than 100 pairs of comparisons, are presented in Additional file 1: Figure S9.

We also directly compared the two groups of cells within a single test (7.75 vs 6.5). This can easily be achieved by assuming a shared splicing ratio *ψ* across all cells in a condition. Mathematically, this is equivalent to multiplying the likelihood terms associated with each cell, or in practice pooling the reads from different cells. While this achieves higher power (see the diamond dot in Fig. 4b), it loses the considerable amount of cell-to-cell heterogeneity highlighted by the single-cell analysis. It would be interesting to explore a more refined way of partial pooling within the hierarchical model [19], or to combine BRIE with scRNA-seq clustering approaches that can identify more homogeneous groups of cells [2].

## Conclusions

Our results demonstrate that BRIE can provide a reliable and reproducible method to quantify splicing levels within single cells. Alternative splicing is a major mechanism for regulation of the transcriptome, and splicing analyses within bulk studies have revealed important associations of splicing with disease. Therefore, the ability to quantify alternative splicing in individual cells would considerably expand the relevance of scRNA-seq technology to investigate variations in RNA processing, and its relevance to diseases. We believe a data-driven informative prior is essential for this task. Directly using bulk RNA-seq methods on scRNA-seq is not a viable route due to the limitations of the technology, an observation that was made earlier [1], which our results confirm. Recent work [20] has addressed the issue of the *detection* of alternative splicing across a population of single cells, but as far as we are aware, BRIE is the first method to be able to *quantify* splicing in individual single cells and to detect differential splicing between individual cells from scRNA-seq data. We notice that, since BRIE focusses on estimating splicing ratios, it is relatively immune to normalization issues, since it is essentially a relative quantification method (see [14] for a compelling demonstration of this property of relative quantification methods).

BRIE provides a flexible framework for modeling and, while sequence features are particularly appealing due to their ease of use and availability, additional side information, such as DNA methylation and chromatin accessibility, could easily be incorporated. Importantly, BRIE is not specific to single-cell RNA-seq technology, and can be of use in any situation where standard quantification is hampered by low coverage.

BRIE’s use of an informative prior enables a smooth trade-off between imputation (at extremely low coverages) and quantification. While this can be a highly effective strategy, it comes at the cost of biasing results at low coverage. In particular, when used with an informative prior learned across several cells, this may lead to underestimating splicing heterogeneity at low coverage. BRIE’s probabilistic formulation, however, brings considerable advantages. In particular, BRIE can be easily combined with other probabilistic modeling strategies aimed at removing confounders such as cell-cycle stage [21], or at estimating pseudo-time [22].

BRIE cannot be deployed on all scRNA-seq protocols, as it assumes that sequenced reads can be distributed along whole transcripts. Naturally, protocols such as CEL-seq or STRT-seq that bias reads towards the ends of the transcript cannot provide information about exon-skipping events that may be very far from the ends of a transcript. We believe that the availability of splicing quantification approaches such as BRIE can, therefore, be an important consideration in experimental design, particularly at a time when single-cell omic technologies are about to start being more routinely employed.

## Methods

### Exon-skipping event annotation

Gene annotations were downloaded from GENCODE human release H22 and mouse release M6. Altogether, 24,957 and 9343 exon-skipping events were extracted from protein-coding genes on human and mouse, respectively. To ensure the high quality of the splicing events, we applied six constraints following two recent studies [11, 23] for filtering:

1. Located on chromosome 1-22 (1-19 for mouse) and X
2. Not overlapped by any other AS exon
3. Surrounding introns are no shorter than 100 bp
4. Length of alternative exon regions between 50 and 450 bp
5. A minimum distance of 500 bp from transcription start site (TSS) or transcription termination site (TTS)
6. Surrounded by AG-GT, i.e., AG-AS.exon-GT

Consequently, 11,478 and 4549 exon-skipping events from human and mouse, respectively, were finally used for this study.

### Feature extraction for Bayesian regression

Following Xiong et al. [11], we extract predictive sequence features from the following seven genomic regions for each exon-skipping event (see cartoon in Fig. 1a): C1 (constitutive exon 1), I1-5ss (300 nt downstream from the 5^′^ splice site of intron 1), I1-3ss (300 nt upstream from the 3^′^ splice site of intron 1), A (alternative exon), I2-5ss (300 nt downstream from the 5^′^ splice site of intron 2), I2-3ss (300 nt upstream from the 3^′^ splice site of intron 2), and C2 (constitutive exon 2).

From these seven regions, four types of splicing regulatory features are defined. First, eight length-related features are included, i.e., log length of C1, A, C2, I1, I2, and the ratio of the log length of A/I1, A/I2 and I1/I2. Second, the motif strengths of the four splice sites, i.e., I1- 5^′^ss, I1- 3^′^ss, I2- 5^′^ss, and I2- 3^′^ss, were calculated from mapping each sequence to its averaged position weight matrix. Here, we considered −4 nt upstream to +6 nt downstream around 5^′^ss (11 nt in total), and from −16 nt to 4 nt for 3^′^ss. Third, we also include evolutionary conservation scores for each of the seven genomic regions, which were calculated by phastCons [24], and are available in the UCSC genome browser. We used the phastCons files in bigWig format with version hg38 for human and mm10 for mouse, where 99 and 59 vertebrate genomes were mapped to the human and mouse genome, respectively. Then the mean conservation scores for the above seven regions were extracted using the bigWigSummary command-line utility. Lastly, 716 short sequences were extracted from the seven regions, including 1-2mers for I1-5ss and I2-3ss (20 sequences each), and 1-3mers for C1, I1-3ss, I2-5ss, and C2 (84 sequences each), and 1-4mers for A (340 sequences). In total, 735 splicing regulatory features were used to predict the exon inclusion ratio in Bayesian regression.

### RNA-seq data and preprocessing

Bulk RNA-seq libraries for the K562 cell line were produced by the ENCODE project [25], and downloaded from the Gene Expression Omnibus (GEO: GSE26284). These were used to validate the prediction performance of the splicing regulatory features on bulk RNA-seq (Additional file 1: Figure S1).

Two single-cell RNA-seq data sets were used to validate the BRIE model. The first data set is from a benchmark study [13], consisting of 96 single-cell RNA-seq libraries from the HCT116 cell line (GEO: GSE51254). These single-cell RNA-seq libraries were prepared with the SMART-seq protocol, and have paired-end reads with a read length of 125 bp. Using a bar code, 48 cells were sequenced per lane, resulting in an average of 2.2 million reads per cell. From the same study, two bulk RNA-seq libraries, each with 31.2 million reads, generated from 1 million HCT116 cells, were also used for comparison. Only reads mapping to alternatively skipped exons and their flanking regions (as described in the previous section) were considered.

To study differential splicing across different cell types, scRNA-seq data produced by the SMART-seq2 protocol from mouse embryo at embryonic day 6.5 and day 7.75 [17] were used. From each of the two groups, 20 individual cells were used, which can be accessed at Array Express (E-MTAB-4079).

All above RNA-seq reads were aligned to the relevant genome reference by HISAT 0.1.6-beta with known splicing junctions.

### Assessing BRIE via a simulation study

Three simulations were conducted to assess BRIE’s performance in quantifying isoforms with low coverages, detecting differential splicing, and imputing splicing in drop-out cases. All synthetic reads were generated by the Spanki simulator [26], while we provide Python wraps to run the simulations easily, which are publicly available in the BRIE GitHub repository.

First, we assessed the robust performance of BRIE in very low coverage on 11,478 human exon-skipping events. We assume that the *ψ* value follows a logitNormal distribution with mean *μ*=0 and *σ*=3, i.e., $\text {\texttt {logit}}(\psi) \sim \mathcal {N}(0, 3.0)$, as presented in Additional file 1: Figure S2, which is like that in the ENCODE K562 cell line. We set all splicing events at the same sequencing coverage, by fixing RPK in each experiment. Finally, five different coverage levels are used, including RPK=25 (very low, but comparable to an average covered gene in a scRNA-seq experiment), RPK=50,RPK=100,RPK=200,and RPK=400.

For generating a feature to learn an informative prior, we added Gaussian noise to the output *ψ* values from the Spanki simulator in its logit format, and ensured a Pearson’s correlation coefficient of 0.8 between the feature and the truth, as shown in Additional file 1: Figure S2. This correlation is like that achieved by supervised learning in a human data set (see Additional file 1: Figure S1). By contrast, five uniformly distributed random features were used to learn a null prior (i.e., a random prior), which is named BRIE.Null.

Second, we tested the power of BRIE in detecting differential splicing events on 400 random mouse exon-skipping events with length ranging from 300 to 800 bp. Eight categories of *ψ* from 0.1 to 0.9 (except 0.5) were equally distributed over the 400 splicing events, and opposite *ψ* values were assigned to two conditions, e.g., *ψ*=0.1 in condition 1 and *ψ*=0.9 in condition 2. Then, the prior was set by the same procedure as the first simulation.

Third, we mimicked the drop-out situation on 11,478 human exon-skipping events, and studied the imputation of BRIE in drop-out cases. We looked at one bulk RNA-seq library and 96 single-cell libraries of HCT116 cell lines [13], and focussed only on the splicing events that are expressed in the bulk cells (FPKM>0). We define the drop-out events as those splicing events that are expressed in the bulk cells (FPKM>0) but not in a given single cell (FPKM=0). We further define the drop-out rate of a single cell as the fraction of drop-out events in this cell, and the drop-out probability of a skipping event as the fraction of its drop-out in 96 cells. Distributions of the drop-out rates and the drop-out probabilities are shown in Additional file 1: Figure S4.

Given an expression profile (e.g., FPKM or transcripts per million (TPM)) *Z* from a bulk library and a profile of drop-out probability calculated from a group of single cells (e.g., the 96 cells here), we simulated the RPK for each isoform (or transcript) as follows. For each isoform *k*, we generate a binary variable *I*_*k*_, i.e., either 0 or 1, following a binomial distribution with the mean as its corresponding drop-out probability. Then each isoform expression level for the simulated single cell is *α* *I*_*k*_ *Z*_*k*_, where coefficient *α* is included to ensure a given number of total reads. If one wants a different overall drop-out rate but keep the similarity of the drop-out probability profile, an intercept will be added to the drop-out probability in its logit space. In the simulation of drop-outs, the 735 sequence features from real data were used to learn the informative prior. We take the mean of the learned prior as the imputed *ψ* for those drop-out events.

### BRIE model for isoform estimates

Here, we formally define the BRIE statistical model. We consider exon inclusion and exclusion as two different isoforms. We start by reviewing the mixture modeling framework for isoform quantification, introduced in MISO [8]. The likelihood of isoform proportions *ψ*_*i*_ for observing *N*_*i*_ reads $R_{i,1:N_{i}}$ in splicing event *i*, can be defined as follows

1 $$ P\left(R_{i,1:N_{i}}|\Psi_{i}\right) = \prod_{n=1}^{N_{i}} \sum_{I_{in}=1}^{2} P\left(R_{in}|I_{in}\right) P(I_{in}| \psi_{i}),  $$

where the latent variable *I*_*in*_ denotes read identity, i.e., where the isoform read *n* in cell *i* came from. For bulk RNA-seq methods like MISO [8] and DICEseq [9], the conditional distribution of the read identity *I*_*in*_|*ψ*_*i*_ is assumed to be a multinomial distribution, and the prior distribution over *ψ*_*i*_ is taken to be an uninformative uniform distribution (suitably adjusted to reflect the potentially different isoform lengths). The pre-computed term *P*(*R*_*in*_|*I*_*in*_) encodes the probability of observing a certain read coming from a specific isoform *I*_*in*_. Bulk methods then proceed usually by adopting a Markov-chain Monte Carlo strategy to sample from the posterior distribution of the *ψ*_*i*_ variables.

BRIE enhances the mixture model approach by combining it with a Bayesian regression module to automatically learn an informative prior distribution by considering sequence features. First, we use a logit transformation of *ψ*_*i*_, i..e, *y*_*i*_ = logit(*ψ*_*i*_). We then model the transformed exon inclusion ratio *y*_*i*_ as a linear function of a set of *m* covariates $X \in \mathbb {R}^{m}$ (here the covariates are the sequence features described previously): *y*_*i*_=*W* ^⊤^ *X*+*ε*_*i*_, where *W* is a vector of weights shared by all samples and *ε*_*i*_ follows a zero-mean Gaussian distribution. All exon-skipping events are independently modeled with shared *W* parameters.

Here, we use a conjugate Gaussian prior for the weights, i.e., $W \sim \mathcal {N}(0, \Lambda ^{-1}) $, with a common choice of *Λ*=*λ* **I**, for a positive scalar parameter *λ*. A graphical representation of the full model is shown in Additional file 1: Figure S10, and the full posterior is as follows (omitting the cell index for simplicity),

2 $${} \begin{aligned} &P(W, \sigma, \boldsymbol{\Psi}\vert \mathbf{X}, \mathbf{R}) \propto\\ &P(W|\lambda)\! \prod_{k=1}^{K}\! \left\{\! P\left(\Psi_{k}|X_{k}, W, \sigma\right)\! \prod_{n=1}^{N_{k}} \sum_{I_{n}^{k}=1}^{2} P\!\left(\!R_{n}^{k}|I_{n}^{k}\right) P\left(I_{n}^{k}| \Psi_{k}\!\right)\! \right\}\!. \end{aligned}  $$

### Inference in BRIE

As shown above, BRIE involves the whole set of exon-skipping events, thus there are thousands of parameters to infer jointly, which can lead to very high computational costs that are not easily distributed. Therefore, we introduce an approximate method to learn *ψ* and *W* alternately. Also, to alleviate the computational burden, there is an option to merge reads from all cells to learn parameters. For simplicity, we set *λ* empirically, using the value *λ*=0.1, which gave the best predictive performance on tests on ENCODE data. Then, we collapse *W* and *σ* by taking their expected value in Bayesian regression given a set of *ψ*, i.e., *W*=(**X** ^⊤^ **X**+*σ* ^2^ *Λ*)^−1^ **X** ^⊤^ **Y** and *σ*=std(**Y**−*W* ^⊤^ **X**). At a single exon-skipping event level, we used an adaptive Metropolis–Hastings sampler to sample *Ψ*, where a univariate Gaussian distribution is used for proposal with adaptive variance, i.e., *η*=2.38×std(*y* ^(1:*m*)^). At this step, we could run short parallel Markov chain Monte Carlo chains on multiple events to alleviate computational costs, for example *h*=50 steps if the total iteration is *n*×*h*=1000. The pseudocode for sampling from the (approximate) posterior distribution of *Ψ* is given in Algorithm 1. Also, this model supports fixed *W* and *σ*, which can be learned from other data sets, e.g., bulk RNA-seq, in which case lines 3 and 5 are turned off in Algorithm 1. The convergence of the sampling is diagnosed using the Geweke diagnostic *Z* score; in our experiments 1000 burn-in steps appeared to be sufficient in all cases.



BRIE then outputs an approximate posterior distribution on the *ψ* values as well as the learned regression weights. BRIE offers functionality to visualize both such posterior distributions as histograms (Fig. 4c) and learned weights as heat maps (Additional file 1: Figure S11 for 19 sequence-related features).

In terms of computational efficiency, on a small sever (48 CPUs and 64 GB memory) using 20 CPUs, BRIE could finish a transcriptome-wide splicing quantification for a human cell (11,478 events) in 5 min, and for a mouse cell (4549 events) in 2 min. This running time is a linear function of the number of cells (learning separate priors for different cells), and can be reduced by using more CPUs.

### Detection of differential splicing using Bayes factors

The BF [27] is a posterior odds in favor of a hypothesis relative to another, and is also able to detect whether splicing in two cells or conditions is different or not.

To detect differential splicing between two cells (or cell groups), *A* and *B*, *δ*=*Ψ*_*A*_−*Ψ*_*B*_, we introduce a null hypothesis (*H*_0_) as *δ*≈0, and the alternative hypothesis (*H*_1_) as *δ*≉0. Here, *D* is the data used to sample the posterior of *Ψ* in two cells. Then, the BF in favor of the alternative hypothesis on observing data *D* is defined as

3 $$ \text{\texttt{BF}} = \frac{P(H_{1}|D)}{P(H_{0}|D)} = \frac{P(D|H_{1})P(H_{1})}{P(D|H_{0})P(H_{0})}.  $$

As usual, we assume that both hypotheses have the same prior, i.e., *P*(*H*_1_)=*P*(*H*_0_), and we can clearly see that *P*(*D*|*H*_0_)=*P*(*D*|*δ*≈0,*H*_1_). Therefore, by taking the Savage–Dickey density ratio [28], we can simplify the calculation of BF as follows,

4 $$ \begin{aligned} \text{\texttt{BF}} & = \frac{P(D|H_{1})}{P(D|\delta \approx 0,H_{1})} \\ & = \frac{P(\delta \approx 0|H_{1})}{P(\delta \approx 0|D,H_{1})} \\ &= \frac{P(-\epsilon < \delta < \epsilon|H_{1})}{P(-\epsilon < \delta < \epsilon|D,H_{1})}, \end{aligned}  $$

where *ε* can be set as 0.05.

As BRIE samples *Ψ*_*A*_ and *Ψ*_*B*_ following their posteriors, the distribution of *P*(*δ*|*D*,*H*_1_) can be readily approximated by empirically re-sampling *Ψ*_*A*_−*Ψ*_*B*_. With a set of re-sampled *δ*_1:*M*_, we take the proportion of |*δ*_*i*_|<*ε* as the posterior probability *P*(−*ε*<*δ*<*ε*|*D*,*H*_1_). Similarly, we could sample a set of $\hat \Psi _{A}$ and $\hat \Psi _{B}$ following their prior distributions, and use the same procedure to approximate the prior probability *P*(−*ε*<*δ*<*ε*|*H*_1_). When comparing two cell groups, one can multiply the individual likelihoods (with shared *ψ* values). This, however, is equivalent to pooling reads across different cells, and will lose the quantification of cell-to-cell heterogeneity.

## Additional files

Additional file 1 Figures S1–S11. (PDF 1940 kb)

Additional file 2 Tables S1 and S2. (XLSX 32.1 kb)

## Abbreviations

BF: Bayes factor
RPK: reads per kilo-base
scRNA-seq: single-cell RNA-seq

## References

[1] Grün D, van Oudenaarden A (2015). Design and analysis of single-cell sequencing experiments. Cell.

[2] Grün D, Lyubimova A, Kester L, Wiebrands K, Basak O, Sasaki N (2015). Single-cell messenger RNA sequencing reveals rare intestinal cell types. Nature.

[3] Shalek AK, Satija R, Shuga J, Trombetta JJ, Gennert D, Lu D (2014). Single cell RNA seq reveals dynamic paracrine control of cellular variation. Nature.

[4] Patel AP, Tirosh I, Trombetta JJ, Shalek AK, Gillespie SM, Wakimoto H (2014). Single-cell RNA-seq highlights intratumoral heterogeneity in primary glioblastoma. Science.

[5] Brennecke P, Anders S, Kim JK, Kołodziejczyk AA, Zhang X, Proserpio V (2013). Accounting for technical noise in single-cell RNA-seq experiments. Nat Methods.

[6] Wang ET, Sandberg R, Luo S, Khrebtukova I, Zhang L, Mayr C (2008). Alternative isoform regulation in human tissue transcriptomes. Nature.

[7] Trapnell C, Williams BA, Pertea G, Mortazavi A, Kwan G, Van Baren MJ (2010). Transcript assembly and quantification by RNA-seq reveals unannotated transcripts and isoform switching during cell differentiation. Nat Biotechnol.

[8] Katz Y, Wang ET, Airoldi EM, Burge CB (2010). Analysis and design of RNA sequencing experiments for identifying isoform regulation. Nat Methods.

[9] Huang Y, Sanguinetti G (2016). Statistical modeling of isoform splicing dynamics from RNA-seq time series data. Bioinformatics.

[10] Liu P, Sanalkumar R, Bresnick EH, Keleş S, Dewey CN (2016). Integrative analysis with ChIP-seq advances the limits of transcript quantification from RNA-seq. Genome Res.

[11] Xiong HY, Alipanahi B, Lee LJ, Bretschneider H, Merico D, Yuen RK (2015). The human splicing code reveals new insights into the genetic determinants of disease. Science.

[12] Bray NL, Pimentel H, Melsted P, Pachter L (2016). Near-optimal probabilistic RNA-seq quantification. Nat Biotechnol.

[13] Wu AR, Neff NF, Kalisky T, Dalerba P, Treutlein B, Rothenberg ME (2014). Quantitative assessment of single-cell RNA sequencing methods. Nat Methods.

[14] Qiu X, Hill A, Packer J, Lin D, Ma YA, Trapnell C (2017). Single-cell mRNA quantification and differential analysis with census. Nat Methods.

[15] Barrass JD, Reid JE, Huang Y, Hector RD, Sanguinetti G, Beggs JD (2015). Transcriptome-wide RNA processing kinetics revealed using extremely short 4tU labeling. Genome Biol.

[16] Shen S, Park JW, Lu Z-X, Lin L, Henry MD, Wu YN (2014). rMATS: robust and flexible detection of differential alternative splicing from replicate RNA-seq data. Proc Natl Acad Sci.

[17] Scialdone A, Tanaka Y, Jawaid W, Moignard V, Wilson NK, Macaulay IC (2016). Resolving early mesoderm diversification through single-cell expression profiling. Nature.

[18] Duymich CE, Charlet J, Yang X, Jones PA, Liang G (2016). DNMT3B isoforms without catalytic activity stimulate gene body methylation as accessory proteins in somatic cells. Nat Commun.

[19] Glaus P, Honkela A, Rattray M (2012). Identifying differentially expressed transcripts from RNA-seq data with biological variation. Bioinformatics.

[20] Welch JD, Hu Y, Prins JF (2016). Robust detection of alternative splicing in a population of single cells. Nucleic Acids Res.

[21] Buettner F, Natarajan KN, Casale FP, Proserpio V, Scialdone A, Theis FJ (2015). Computational analysis of cell-to-cell heterogeneity in single-cell RNA sequencing data reveals hidden subpopulations of cells. Nat Biotechnol.

[22] Campbell K, Yau C. Ouija: Incorporating prior knowledge in single-cell trajectory learning using Bayesian nonlinear factor analysis. bioRxiv 060442. 2016.

[23] Curado J, Iannone C, Tilgner H, Valcárcel J, Guigó R (2015). Promoter-like epigenetic signatures in exons displaying cell type-specific splicing. Genome Biol.

[24] Pollard KS, Hubisz MJ, Rosenbloom KR, Siepel A (2010). Detection of nonneutral substitution rates on mammalian phylogenies. Genome Res.

[25] ENCODE Project Consortium (2012). An integrated encyclopedia of DNA elements in the human genome. Nature.

[26] Sturgill D, Malone JH, Sun X, Smith HE, Rabinow L, Samson ML (2013). Design of RNA splicing analysis null models for post hoc filtering of *Drosophila* head RNA-seq data with the splicing analysis kit (Spanki). BMC Bioinform.

[27] Kass RE, Raftery AE (1995). Bayes factors. J Am Stat Assoc.

[28] Verdinelli I, Wasserman L (1995). Computing Bayes factors using a generalization of the Savage–Dickey density ratio. J Am Stat Assoc.

