# Supplementary material for: BRIE: transcriptome-wide splicing quantification in single cells
Source: Genome Biol. 2017 Jun 27;18:123. doi: 10.1186/s13059-017-1248-5 (PMC5488362; doi:10.1186/s13059-017-1248-5)
Supplement: Supplementary file 1 — Figures S1–S11. (PDF 1940 kb) [file 13059_2017_1248_MOESM1_ESM.pdf]

Supplementary figures for "Transcriptome-wide splicing quantification in single cells"

Yuanhua Huang[1], and Guido Sanguinetti[1,2,*]

[1]School of Informatics, University of Edinburgh, Edinburgh EH8 9AB, UK; [2]Centre for Synthetic and Systems Biology (SynthSys), University of Edinburgh, Edinburgh, EH9 3BF, UK
[*]To whom correspondence should be addressed. Email: G.Sanguinetti@ed.ac.uk

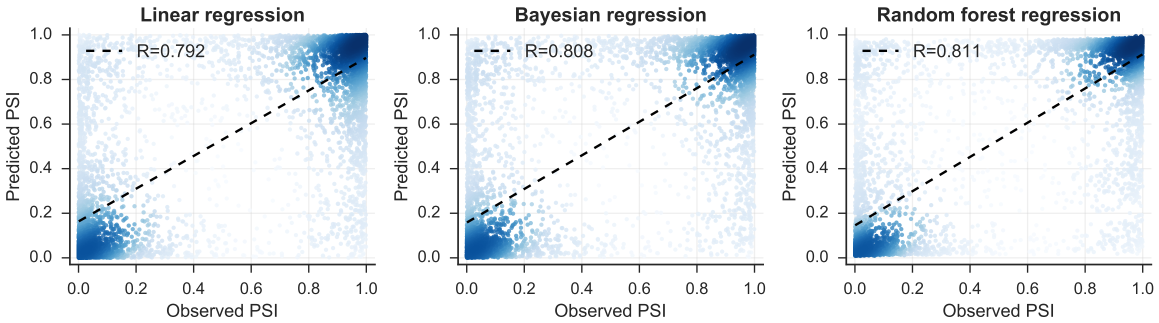

Figure S1: Sequence features are predictive for exon-skipping events. 735 sequence features are used to predict inclusion ratios of 6,922 out of 11,478 skipping exon (95CI$<$ 0.3) on human K562 cell line, with ordinary linear regression (left panel), Bayesian regression (middle panel), and random forest regression (right panel).

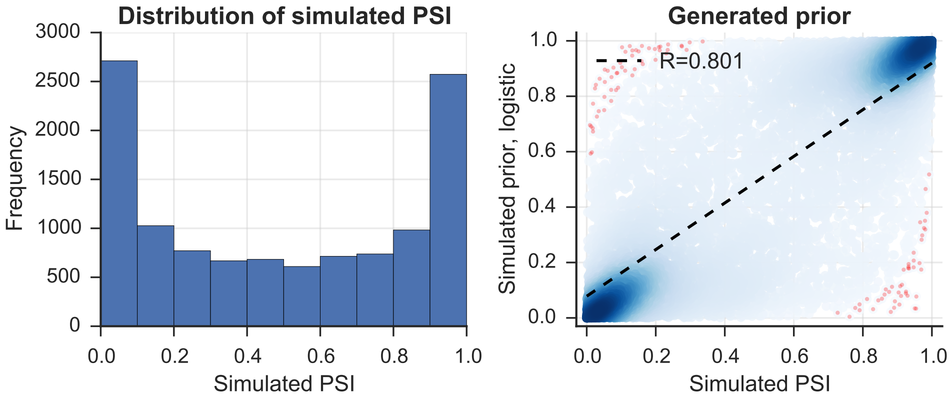

Figure S2: The settings for simulation of exon inclusion ratio and corresponding prior. Left panel: the distribution of simulated inclusion ratio ψ, which follows a logit-normal distibution with mean $\mu = 0$ and variance $\theta^2 = 3^2$. Right panel: the correlation between the generated feature (for learning prior) and the input truth for simulation.

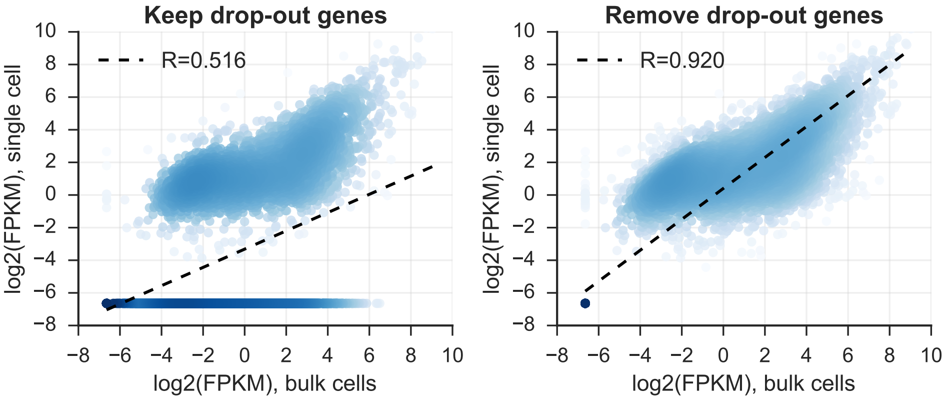

Figure S3: Effects of drop-out in single-cell RNA-seq. Scatter plot of log2(FPKM) of exon-triplets in human HCT116 cell line between bulk cells and a single cell. The drop-out genes are defined as thoese with $FPKM > 0$ in bulk cells and $FPKM = 0$ in the single cell. Left panel: keep the drop-out genes; Right panel: remove drop-out genes. The Pearson's correlation coefficient increases from 0.516 to 0.920 after remove drop-out genes.

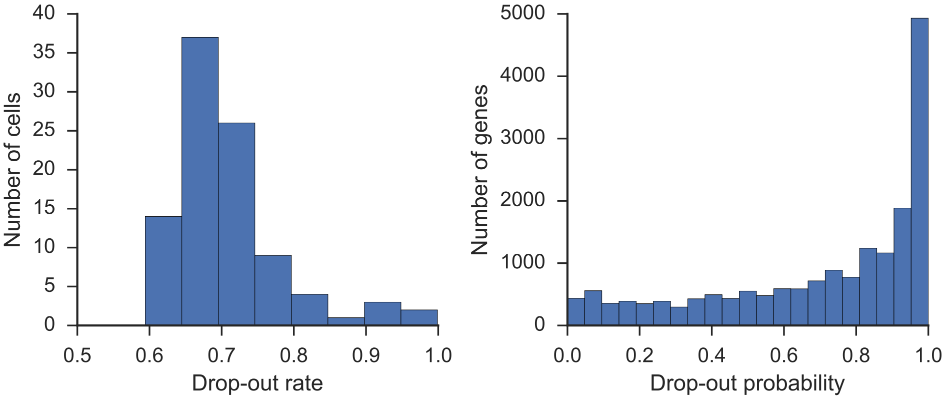

Figure S4: The distribution of drop-out rate and drop-out probability of exon-triplets. Left panel: drop-out rate distribution across 96 human HCT116 cells. Drop-out rate calculate by the fraction of expressed exon-triplets in bulk cells that have no reads in single cells. Right panel: drop-out probability of each gene, which is calculated by the frequency of its drop-out in 96 cells.

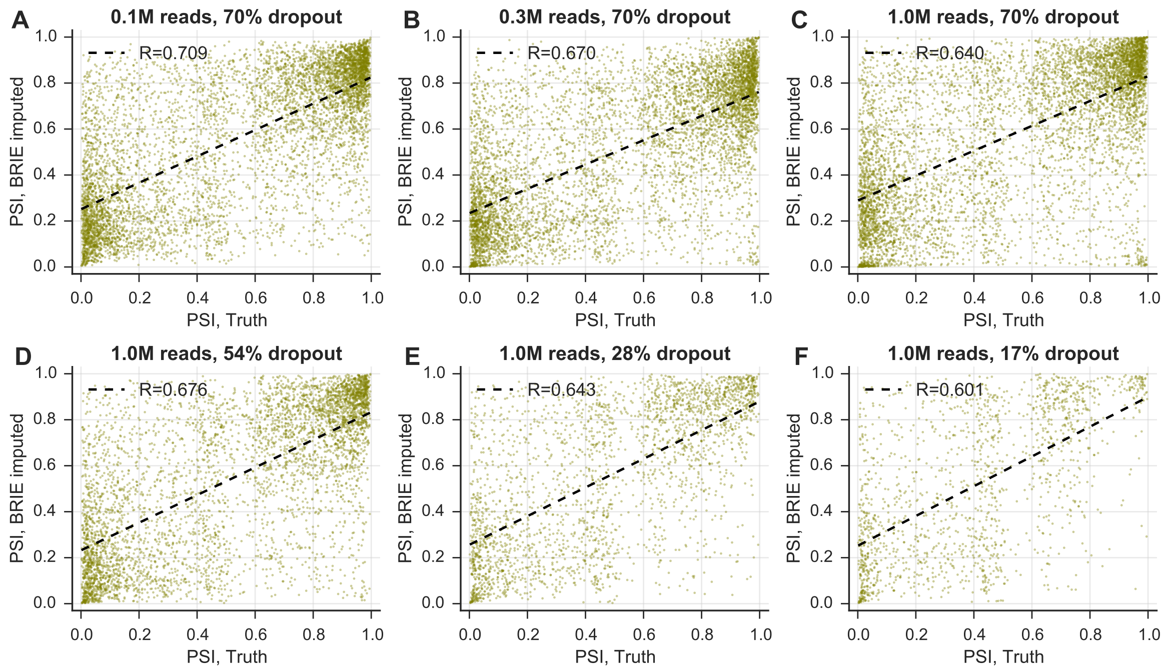

Figure S5: Imputation of exon inclusion ratio for drop-out genes with simulated data. Following the expression profile of bulk cells and the drop-out probability of 96 single cells, the RNA-seq reads library is generated with a given number of total reads and an overall drop-out rate. The true inclusion ratio ψ is the value in bulk RNA-seq and also the input for the simulator. The features are the same 735 genetic features for real data set.

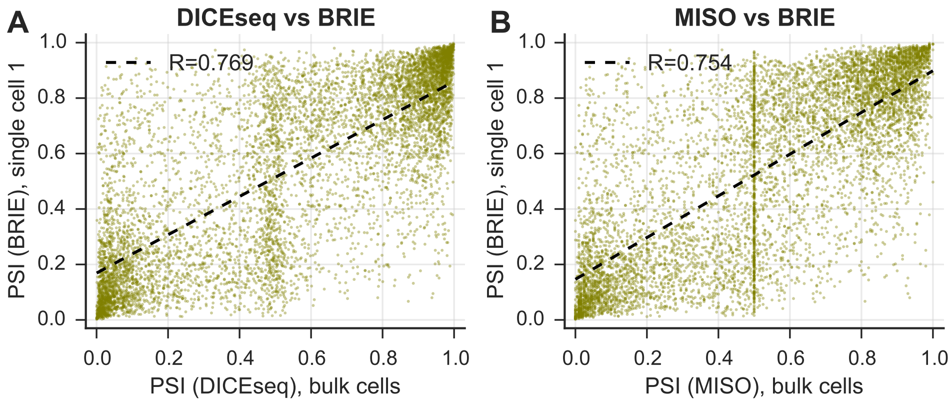

Figure S6: Scatter plots of exon inclusion ratio estimates from HCT116 cells. (A) Bulk cells by DICE-seq and single cell by BRIE. (B) Bulk cells by MISO and single cell by BRIE.

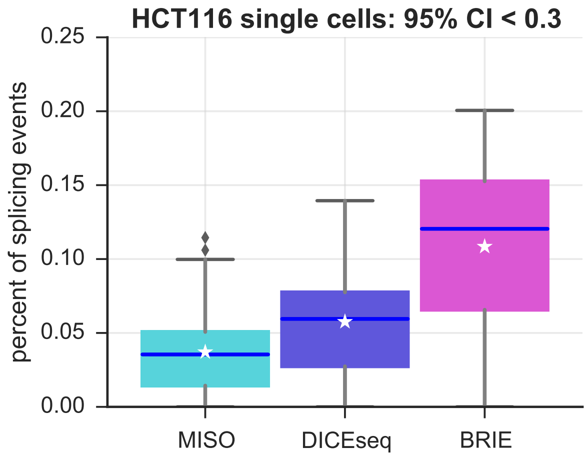

Figure S7: Box plot of percentage of splicing events that have 95% confidence interval <0.3 in 96 HCT116 cells. Three methods are used: MISO, DICEseq, BRIE.

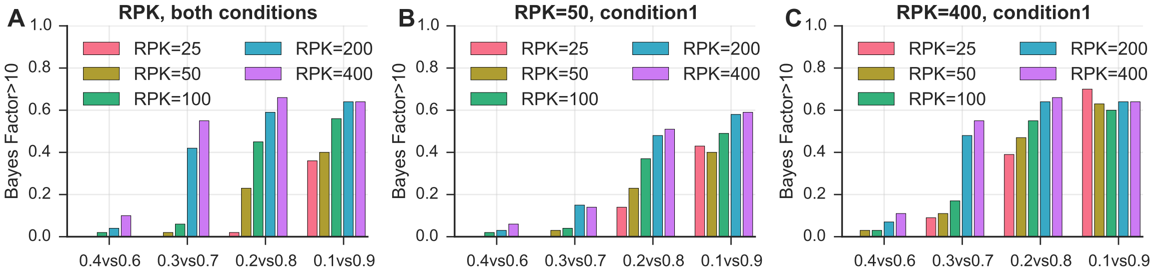

Figure S8: Percentage of genes with $BayesFactor > 10$ by comparing two conditions in different coverages and ψ values. (A) Coverages for both conditions are the same, ranges from RPK=25 to RPK=400. (B) RPK fixed as 50 in condition 1. (C) RPK fixed as 400 in condition 1.

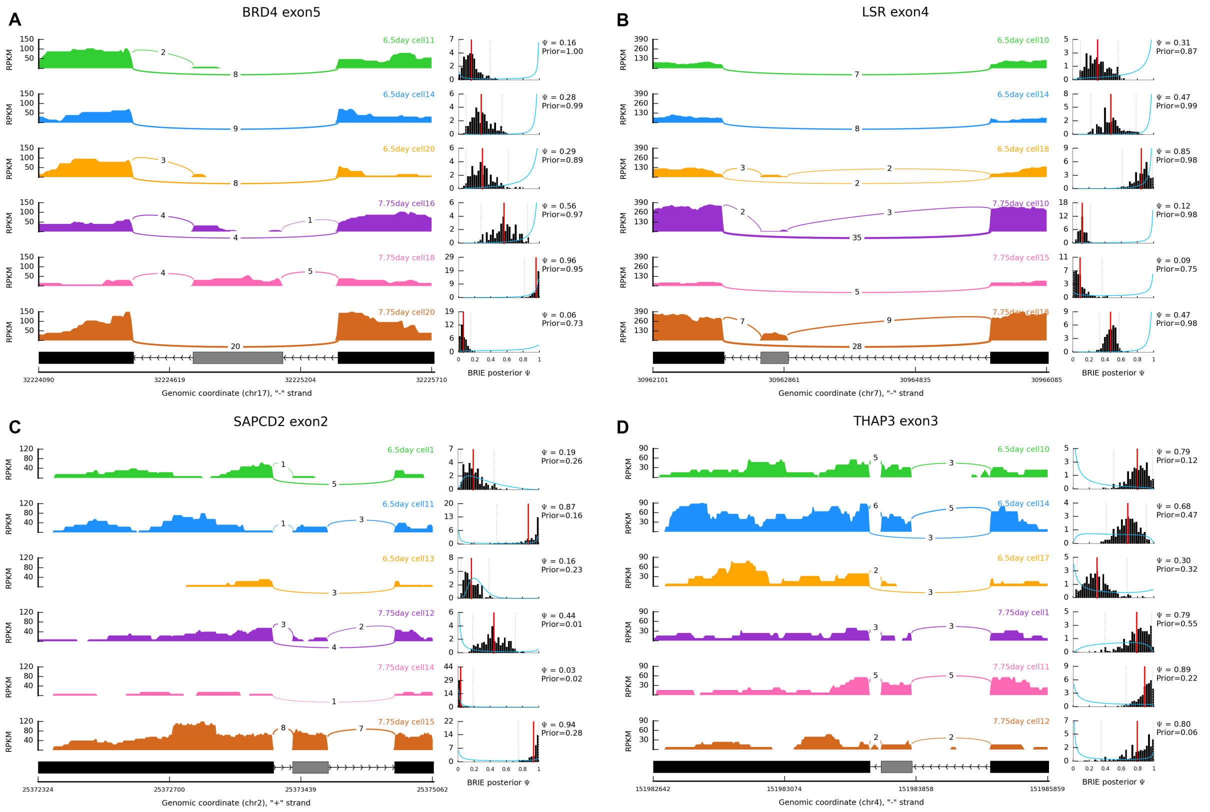

Figure S9: Four more example events with high splicing variation between 6.5 days and 7.75 days. The format of the figure is the same as Fig 4c.

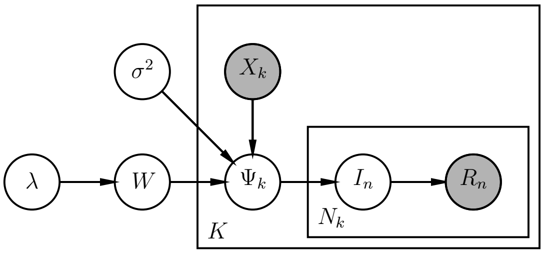

Figure S10: Graphical representation of the BRIE model, which combines a Bayesian regression to learn an informative prior and a mixture model of RNA-seq reads (likelihood). The left part is the Bayesian regression that uses a set of features X_k to predict the ratios Ψ_k for K splicing events. The right part is a mixture model giving the likelihood of observed RNA-seq reads given the splicing ratio. For each splicing event, the observation of N_k reads $\mathbf{R} = (R_1, ..., R_N)$ are considered as N_k conditionally independent events, which depend on the originating isoform I_n, whose probability depends on the splicing ratio Ψ_k. Shaded nodes represent observed variables.

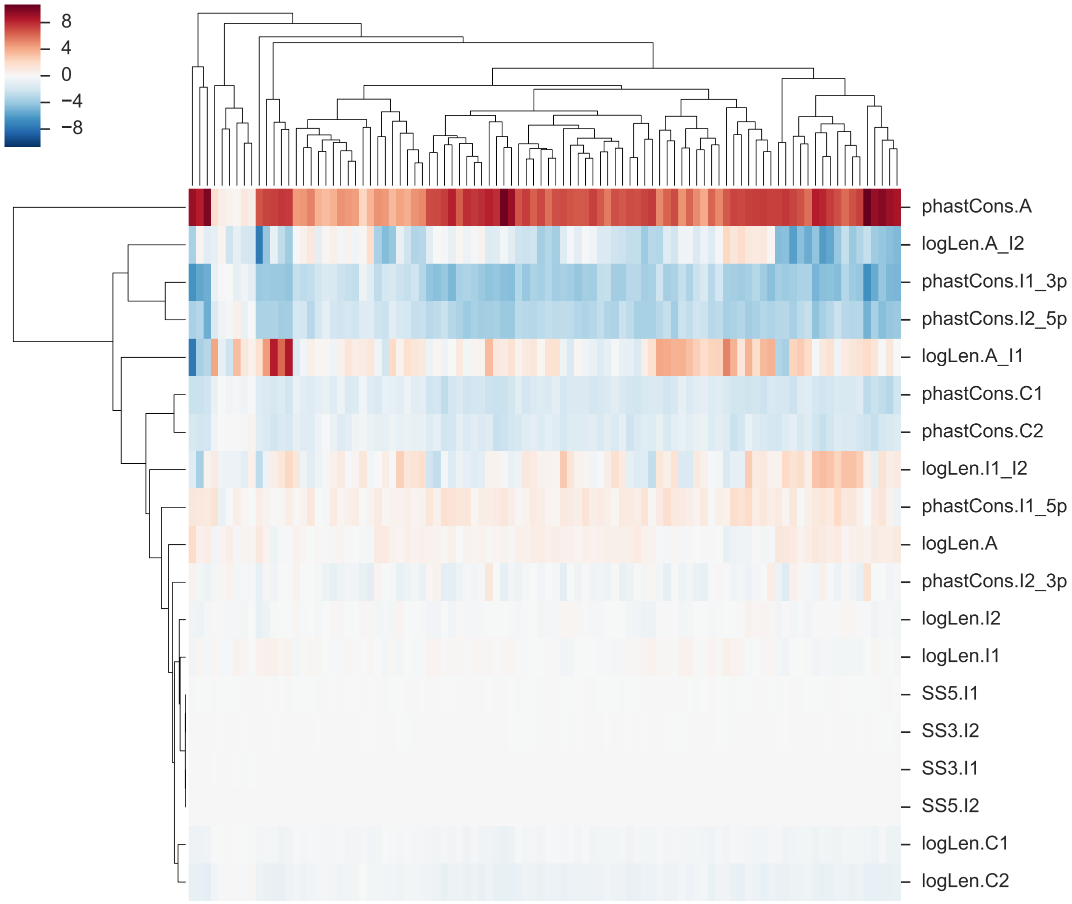

Figure S11: Heatmap of weights of 19 sequence related features, learned by BRIE in 96 cells. The x-axis is 96 cells, and the y-axis is 19 features.
